# Bap, a Biofilm Matrix Protein of *Staphylococcus aureus* Prevents Cellular Internalization through Binding to GP96 Host Receptor

**DOI:** 10.1371/journal.ppat.1002843

**Published:** 2012-08-02

**Authors:** Jaione Valle, Cristina Latasa, Carmen Gil, Alejandro Toledo-Arana, Cristina Solano, José R. Penadés, Iñigo Lasa

**Affiliations:** 1 Laboratory of Microbial Biofilms, Idab-Universidad Pública de Navarra-CSIC-Gobierno de Navarra, Pamplona, Spain; 2 Instituto de Biomedicina de Valencia (IBV-CSIC), Valencia, Spain; University of Washington, United States of America

## Abstract

The biofilm matrix, composed of exopolysaccharides, proteins, nucleic acids and lipids, plays a well-known role as a defence structure, protecting bacteria from the host immune system and antimicrobial therapy. However, little is known about its responsibility in the interaction of biofilm cells with host tissues. *Staphylococcus aureus*, a leading cause of biofilm-associated chronic infections, is able to develop a biofilm built on a proteinaceous Bap-mediated matrix. Here, we used the Bap protein as a model to investigate the role that components of the biofilm matrix play in the interaction of *S. aureus* with host cells. The results show that Bap promotes the adhesion but prevents the entry of *S. aureus* into epithelial cells. A broad analysis of potential interaction partners for Bap using ligand overlayer immunoblotting, immunoprecipitation with purified Bap and pull down with intact bacteria, identified a direct binding between Bap and Gp96/GRP94/Hsp90 protein. The interaction of Bap with Gp96 provokes a significant reduction in the capacity of *S. aureus* to invade epithelial cells by interfering with the fibronectin binding protein invasion pathway. Consistent with these results, Bap deficient bacteria displayed an enhanced capacity to invade mammary gland epithelial cells in a lactating mice mastitis model. Our observations begin to elucidate the mechanisms by which components of the biofilm matrix can facilitate the colonization of host tissues and the establishment of persistent infections.

## Author Summary

*Staphylococcus aureus* is a pathogen responsible for a wide variety of infections, some of which become chronic due to the capacity of this bacteria to form multicellular communities that grow embedded in a self-produced extracellular matrix, referred to as biofilms. Numerous evidences have demonstrated that growing in the biofilm protects bacteria from the immune system and antimicrobial treatments. However, less attention has been paid to the role that the biofilm extracellular matrix plays in the interaction with host cells. Here, we investigate this issue through the use of the proteinaceous biofilm matrix assembled by the Bap protein as a model. Our results show that the Bap biofilm matrix triggers the adhesion to epithelial cells. After adhesion, Bap binds directly to the host receptor Gp96 and this interaction inhibits the entry of the bacteria into the cells by interfering with the fibronectin-binding protein mediated invasion pathway. As a result, the expression of Bap decreased cell invasion and increased bacterial persistence in lactating mice mammary glands. Thus, our findings revealed a dual role for the Bap-dependent biofilm matrix during the establishment of persistent infections, promoting adhesion of *S. aureus* to epithelial cells and impairing host cell invasion.

## Introduction

*Staphylococcus aureus* is a regular commensal of the skin of animals and human population and it persistently colonizes the anterior nares of around 25% of human adults [1]. *S. aureus* is harmless in these locations, but it turns into an extremely threatening pathogen when it traverses the epithelial barrier and gains access to internal tissues from where it can infect almost any organ and cause a broad spectrum of infections including abscesses, pneumonia, endocarditis, osteomyelitis, sepsis and infections associated with foreign-body implants [2]. Once in the internal tissue, *S. aureus* remains mainly extracellular, in the interstitial space between the cells [3], [4], where bacteria encounter cellular, humoral and complement compounds of the host innate immune system. To succeed in this environment, *S. aureus* produces a large variety of virulence factors that mediate cell and tissue adhesion (surface proteins), contribute to tissue damage and spreading (proteases, coagulase, DNAse, lipases, toxins) and protect bacteria against the host immune defense system (superantigens) [5], [6]. In some cases, *S. aureus* proliferates producing bacterial aggregates that grow encased in a self-produced extracellular polymeric matrix, known as a biofilm [7]–[11]. Based on the susceptibility of the biofilm matrix to the disaggregation with glycoside hydrolases (dispersin B), proteases or DNAse, it is recognized that the *S. aureus* biofilm matrix can be made of exopolysaccharides, proteins and DNA [12]–[17]. The exopolysaccharidic biofilm matrix is composed of a polymer of poly-N-acetyl-β-(1–6)-glucosamine, termed polysaccharide intercellular adhesin (PIA) or poly-N-acetylglucosamine (PNAG) [18]–[21]whereas the proteinaceous biofilm matrix can be assembled with different surface proteins, namely Bap, FnBPs, SasG and Protein A [16], [17], [22]–[26].

The first example of a surface protein able to induce biofilm development was Bap. It is a large protein of 2,276-aminoacids with a series of identical repeats of 86 amino acids that accounts for more than half of the protein [22], and two canonical calcium binding EF-hand motifs able to control Bap functionality in response to the calcium present in the growth media [27]. The *bap* gene in *S. aureus* was initially identified in a mobile pathogenicity island (SaPIbov2) whose mobility depends on the activity of a self-encoded recombinase protein [28]. So far, the *bap* gene has never been found in *S. aureus* human isolates. However, a *bap* ortholog gene is present in the core genome of several coagulase-negative staphylococcal species that are frequent colonizers of human skin [29].All the *S. aureus* strains harbouring the *bap* gene are strong biofilm producers and Bap-mediated biofilm formation process occurs independently of the presence of the PIA/PNAG exopolysaccharide [30].A particularly interesting issue concerning this Bap assembled matrix is that functionally related proteins homologous to Bap exist in many phylogenetically unrelated bacteria including *Enterococcus faecalis*, *Acinetobacter baumanii*, *Pseudomonas aeruginosa*, *Salmonella enteritidis*, *Lactobacillus reuteri*, *Bordetella pertussis* and *Escherichia coli* (for a review see [31]). All the Bap homologous proteins show high molecular weight, contain a core domain of repeats and promote bacterial aggregation and biofilm development.

It is currently clear that matrix production and subsequent biofilm formation is very often associated with the establishment of persistent infections due to the highly resistant nature of the embedded bacteria to both the host immune defences and the antimicrobial therapy. In this respect, several studies have demonstrated that the PIA/PNAG matrix aids *S. aureus* in the evasion of host immune defences by protecting bacteria from macrophage phagocytosis and attenuating host proinflammatory responses [32]–[35].Furthermore, phenol-soluble modulins (PSMs) surfactant peptides secreted to the biofilm matrix of *S. aureus* act as biofilm structuring factors but also have multiple functions in immune system evasion [35], [36].

In addition to its protecting role, it is feasible to envision that the biofilm matrix may mask important bacterial surface antigens, and in this context, the interaction between bacteria inside the biofilm and the host might rely on the specific binding of extracellular matrix components to host cell receptors. Accordingly, various studies have proposed a role of the Bap-mediated biofilm matrixes, including BapA of *S.* Enteritidis, Esp of *E. faecalis*, Lsp of *L. reuteri* and Bap of *A. baumanii*, in the adhesion to host cells [37]–[40]. With regard to Bap of *S. aureus*, we have previously shown that the presence of a Bap-mediated biofilm matrix interferes with the binding of several *S. aureus* adhesins (fibronectin-binding protein and clumping factor) to their targets (fibrinogen and fibronectin) in host tissues [41]. Despite this masking effect caused by the Bap matrix, *S. aureus* strains producing Bap display an enhanced capacity to colonize and persist in the mammary gland [30].However, the underlying molecular mechanisms of the interaction between the Bap related proteins and eukaryotic cells remain unknown.

In this study, we used the staphylococcal Bap mediated matrix as a model to investigate the role that components of the biofilm matrix play in the interaction with host cells. Our results revealed that Bap enhances the adhesion but inhibits the entry of *S. aureus* into the epithelial cells. We also identified a direct interaction between Bap and the Gp96 chaperone protein from host cells. Binding of Bap to Gp96 was responsible for the inhibition of bacterial invasion into nonprofessional phagocytic cells by interfering with the fibronectin-binding protein mediated internalization pathway. Overall, our results reveal new facets of the roles that the biofilm matrix plays during the establishment of persistent infections.

## Results

### Bap promotes adhesion but inhibits the entry of *S. aureus* into epithelial cells

**Figure 1:**
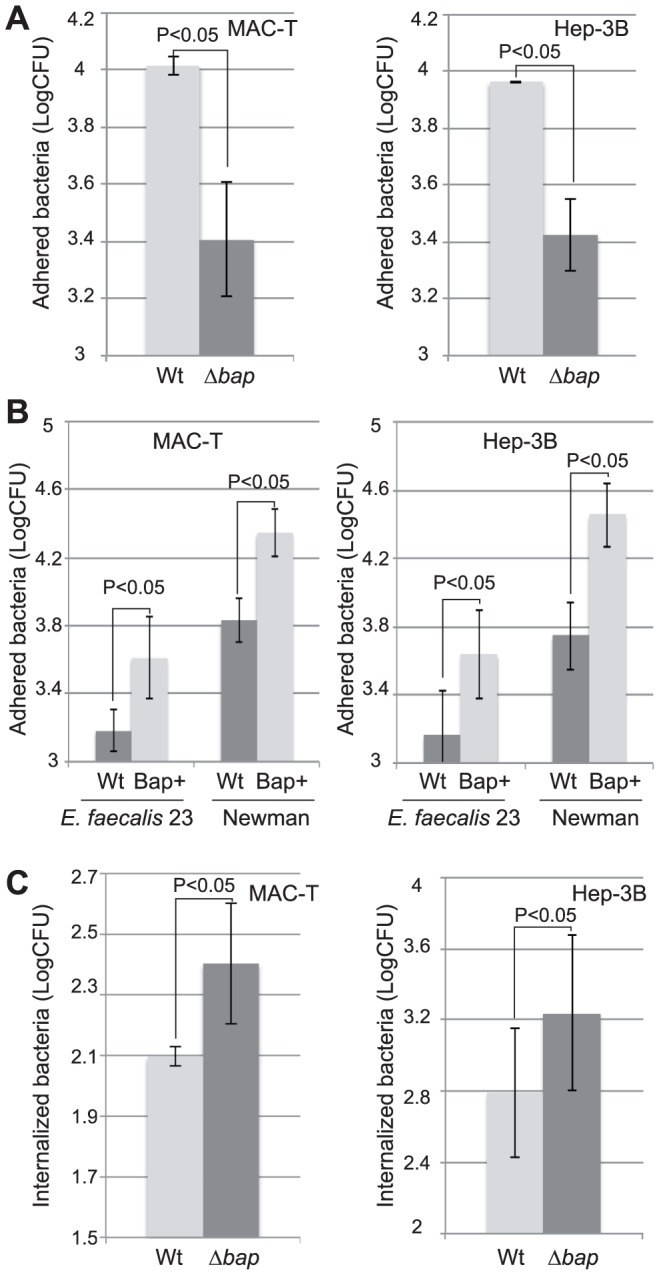
A) Effect of Bap expression in *S. aureus* adherence to epithelial cells. Adhesion of *S. aureus* V329 wild type (Wt) and Δ*bap* mutant to the bovine mammary gland epithelial cells MAC-T and to human hepathocytes Hep-3B. B) Adhesion of *S. aureus* Newman wild type (Wt), *S. aureus* Newman expressing Bap (Bap+), *E. faecalis* 23 wild type (Wt) and *E. faecalis* expressing Bap (Bap+) to MAC-T and Hep-3B epithelial cells. After 1 h of infection, cells were lysed and cell extracts were vigorously vortexed for 2 min. Bacterial adhesion was measured by CFU counts. Increase in adhesion was statistically significant (p value<0,05) in all comparisons shown. C)Effect of Bap expression in *S. aureus* entry into MAC-T and Hep-3B epithelial cells. Bacterial invasion was measured by CFU count after gentamicin assay. Experiments were performed in triplicate and repeated four times for each cell line.

To analyze the involvement of the Bap matrix on the adherence capacity of *S. aureus*, we tested the ability of *S. aureus* V329 strain and Δ*bap* mutant to adhere to two different cell lines, a bovine mammary epithelial (MAC-T)and a human hepatocyte (Hep-3B) cell line. The bacterial inoculum used in cell assays came from an overnight culture in which *S. aureus* V329 strain aggregated at the bottom of the tube and also formed a Bap-dependent biofilm adhered to the glass wall, whereas Δ*bap* mutant grew planktonically (data not shown). The results revealed that the V329 strain adhered 4-fold more efficiently (P<0.05) than the Δ*bap* mutant to both cell lines (Figure 1A). It is worth noting that Bap-negative bacteria also showed a reduce capacity to adhere to a HEK293 cell line, despite the fact that these differences passed unnoticed in a previous study of our group [41].We then compared the adhesion of a natural *bap*-negative strain, *S. aureus* Newman, and its isogenic derivative containing a chromosomal copy of the *bap* gene (*S. aureus* Newman_Bap) [27]. *S. aureus* Newman_Bap showed a5 times (P<0.05) higher capacity to adhere to both MAC-T and Hep-3B cell lines than its parental *bap*-deficient *S. aureus* Newman strain (Figure 1B). These results indicated that the presence of Bap enhances the capacity of *S. aureus* to bind to epithelial cells, but they did not answer the question as to whether the Bap protein was sufficient to promote adhesion. To elucidate this point, the Bap protein was expressed in a heterologous surrogate bacterium, *Enterococcus faecalis* and its adherence capacity was tested. *E. faecalis* producing the Bap protein adhered significantly more efficiently to both MAC-T and Hep-3B cell lines (P<0.05) than the corresponding wild type strain (Figure 1B). Taken together, these results demonstrated that the Bap protein confers the capacity to adhere to epithelial cells.

Next, taking into account that *S. aureus* strain V329 invaded human embryonic kidney cells (HEK293)less efficiently than its corresponding Bap-deficient strain [41],we decided to test whether Bap could also block *S. aureus* entry in our cellular models, MAC-T and Hep-3B cells. Quantification of intracellular bacteria, after invasion assays with *S. aureus* V329 and Δ*bap* revealed that *S. aureus* Δ*bap*, despite its deficiency in the adhesion capacity to epithelial cells, was able to invade more efficiently MAC-T and Hep-3B cells than the wild type strain(Figure 1C). To ensure that inhibition of *S. aureus* entry was due to the presence of Bap rather than to the presence of a biofilm matrix, we compared the invasion capacity of *S. aureus* V329 and Δ*bap* strains complemented with a plasmid carrying the *icaADBC* operon (pSC18) as well as *S. aureus* ISP479r, a strain that constitutively produces large amounts of PIA/PNAG exopolysaccharide, and its isogenic Δ*ica* mutant. The results showed that the presence of the PIA/PNAG matrix does not have any effect on the capacity of bacteria to invade epithelial cells (Supplementary Figure S1).

Taken together, these results showed that the Bap-mediated matrix promotes bacterial adhesion to epithelial cells, and on the other hand, interferes with *S. aureus* cell entry.

### Identification of Gp96 as the ligand for Bap

**Figure 2:**
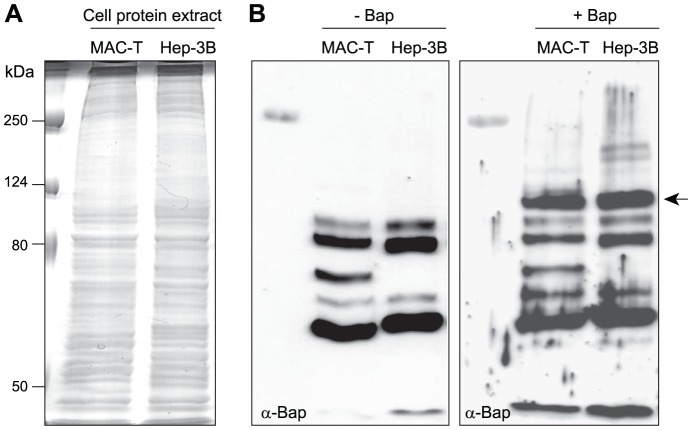
Identification of Gp96 as a Bap cellular receptor by a ligand overlayer assay. A) Total protein extracts from MAC-T and Hep-3B cells were separated on a SDS gel, transferred to a nitrocellulose membrane by western-blotting and probed with/without pure Bap protein (50 µg/ml). B) Bound Bap protein was detected with polyclonal anti-Bap antibodies (α-Bap). A ∼100 kDa band reacting with anti-Bap antibodies is shown by the arrow. This protein was identified by MALDI-TOF analysis of the comigrating band on a Coomasie blue stained gel as Gp96.

We then aimed to identify putative host cell receptors interacting with Bap by means of a ligand overlay approach. MAC-T and Hep-3B total cell extracts were separated by SDS-polyacrylamide gel electrophoresis, transferred onto a nitrocellulose membrane and incubated in the presence of purified recombinant Bap protein containing a 6xHistidine tag replacing the LPXTG motif. Then, proteins that bound specifically to Bap were detected by probing the membrane with an anti-Bap serum. A Coomassie blue stained gel of the total membrane protein profile of each strain is shown for reference (Figure 2A). We focused on Bap-reactive bands that were present in both cell extracts and absent when the membranes were not incubated with the Bap protein (Figure 2B). A prominent band in the range of ∼100 kDa was apparent in both MAC-T and Hep-3B cell extracts. To exclude that the 6xhistidine tag present in the recombinant Bap protein could be responsible for the interaction with the eukaryotic protein, we performed a similar ligand overlayer assay using the unrelated6xHis tagged dispersin protein. As expected, no specific bands reacting with the cell extracts were detected when this protein was used as a bait (data not shown).

To determine the identity of the ∼100 kDa Bap binding protein, the region corresponding to the location of the ∼100 kDa band was excised from a parallel Coomassie stained gel and this sample was subject to trypsin digestion and MALDI-TOF analysis followed by peptide mass fingerprinting that was compared with the human proteome. The results showed that the protein band corresponded to the endoplasmic reticulum chaperone Gp96 (GRP94), a member of the Hsp90 family of molecular chaperones [42], [43].

### Gp96 is expressed at the cell surface of MAC-T and Hep-3B cells

**Figure 3:**
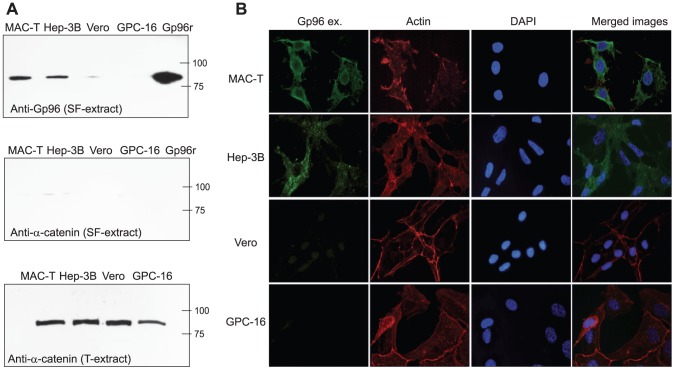
Gp96 is expressed at the cell surface of MAC-T and Hep-3B cells. A) Western-blot analysis of Gp96 expressed at the cell surface. Surface exposed proteins (SF-extract) of MAC-T, Hep-3B, Vero and GPC-16 cells were labeled with sulpho-N-hydroxysuccinimide (NHS) biotin and purified using streptavid in columns. Captured proteins were separated in 10% SDS gel, transferred onto a nitrocellulose membrane and probed with anti-Gp96. To control membrane impermeability, α-catenin was detected from the surface proteins (SF-extract) using anti-α-catenin antibodies, whose specificity was verified using total cell extracts (T-extract). B) Cellular localization of Gp96. The cellular distribution of Gp96 was analyzed by immunofluorescence using nonpermeabilized MAC-T, Hep-3B, Vero and GPC-16 cells labelled with anti-Gp96 (Gp96 ex.), phalloidin (actin) and DAPI.

Although Gp96 is recognized as an endoplasmic reticulum chaperon for Toll-like receptors [44], numerous evidences indicate that it is also expressed on the plasma membrane of different cell types [45]–[55].Thus, we decided to analyze whether Gp96 protein is expressed at the cell surface of MAC-T and Hep-3B cells. As a negative control, we also included in the assay two cell lines (Vero and GPC-16) that have been previously shown to poorly express extracellular Gp96 [52]. Firstly, we confirmed the expression of Gp96 in whole MAC-T and Hep-3B cell extracts by immunoblotting using anti-Gp96 antibodies (data not shown). Secondly, we investigated if Gp96 was localized in the plasma membrane of MAC-T and Hep-3B cells, by means of labelling surface exposed proteins of intact cells using the membrane-impermeable biotinylation reagent sulpho-N-hydroxysuccinimide (NHS) biotin (Pierce). After harvesting the cells, the biotinylated proteins were purified on streptavidine-beads. Upon reduction, the biotinylated proteins were released from the beads and analyzed by western-blot using anti-Gp96 antibodies. The results revealed the presence of Gp96 in the biotinylated protein fraction of MAC-T and Hep-3B cells. Accordingly, a faint band was detected in Vero and GPC-16 cells. In addition, to verify that membrane impermeability was not disrupted during the assay and exclude that cytoplasmic Gp96 could be labeled during the experiment, the biotinylated protein fractions were interrogated using anti-α-catenin antibodies. The absence of α-catenin, an abundant protein in the cytoplasm, confirmed that the biotinylated fraction did not contain cytoplasmic proteins (Figure 3A).

Lastly, we used immunofluorescence staining to localize Gp96 distribution at the cell surface. Nonpermeabilised MAC-T, Hep-3B, Vero and GPC-16 cells were incubated with polyclonal anti-Gp96 followed by incubation with a secondary antibody conjugated with Alexa-488. Then, cells were permeabilized and F-actin was labelled with Alexa-Fluor 647-phalloidin. In agreement with previous results, Gp96 was clearly detected at the cell surface of nonpermeabilized MAC-T and Hep-3B cells whereas it was absent from the cell surface of GPC-16 and Vero cells (Figure 3B).

### Gp96 is a ligand for Bap on intact *S. aureus*

**Figure 4:**
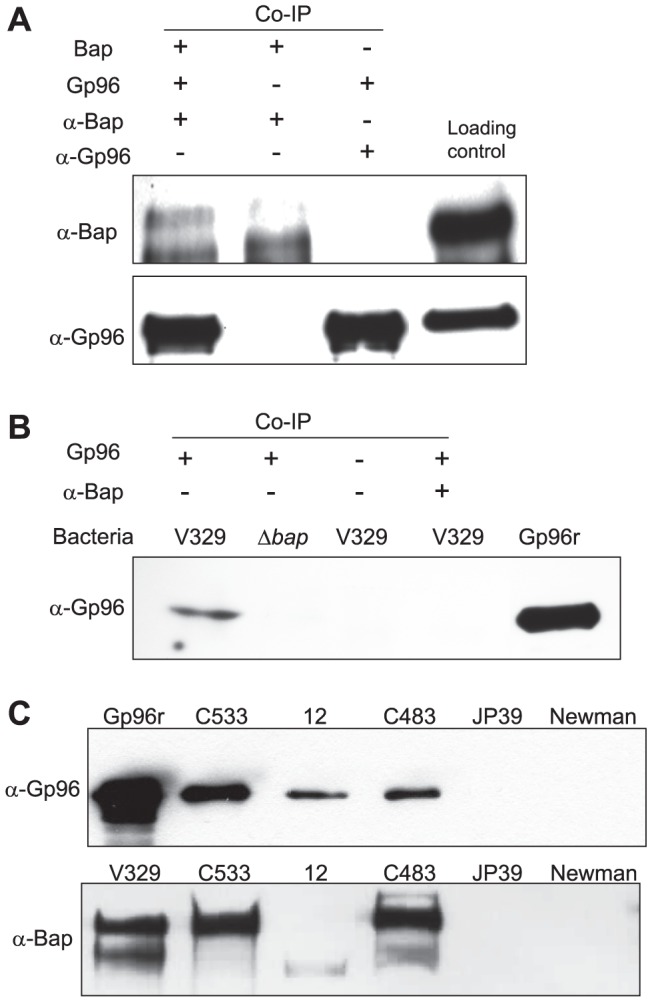
Bap binds the cellular receptor Gp96. A) For the co-immunoprecipitation (Co-IP) recombinant Gp96 was incubated with purified Bap protein and immunoprecipitated with anti-Bap and protein G sepharose beads. In the control assay, the incubation step with Bap or Gp96 was omitted. Immunoprecipitated proteins were separated in 10% SDS gel, transferred onto a nitrocellulose membrane and probed with anti-Gp96 (α-Gp96) or anti-Bap (α-Bap). B) Recombinant Gp96 binds to *S. aureus* expressing Bap. Bacteria expressing Bap (V329) and the Bap deleted mutant (Δ*bap*) were subcultured with 5 µg/ml of recombinant Gp96 in the absence or presence of anti-Bap serum as indicated. Unbound Gp96 was removed by extensive washing and Gp96 bound to bacteria was detected using immunoblot analysis. C) Bap proteins of coagulase negative staphylococci(*S. epidermidis* C533, *S. hyicus* 12, *S. chromogenes* C483) bind Bap. Bacteria were subcultured with 5 µg/ml of recombinant Gp96. Unbound Gp96 was removed by extensive washing and Gp96 bound to bacteria was detected using immunoblot analysis (upper part). Bap expression in *S. epidermidis* C533, *S. hyicus* 12, *S. chromogenes* C483 by immunoblot using polyclonal anti-Bap (α-Bap) serum (lower part).

One caution of the ligand overlay assay is that proteins presented in non-native conformations may interact in artificial ways with a ligand and lead to the detection of “false positive” interactions. Several lines of evidences were used to independently verify that Gp96 is a ligand of Bap. First, we assessed the interaction of purified Bap with recombinant Gp96 by coimmunoprecipitation using anti-Bap polyclonal antibodies, and as a result, we found a band corresponding to Gp96 after immunoprecipitation of the complex (Figure 4A). Second, we analyzed the binding of Gp96 to live, intact *S. aureus* bacteria. For that, *S. aureus*V329 (Bap+) and it isogenic *S. aureus* Δ*bap* mutant were incubated with recombinant Gp96 protein. Binding of Gp96 to *S. aureus* was detected by western-blot using monoclonalanti-Gp96 antibodies. As shown in figure 4B, the presence of Gp96 was only detected in cell extracts of *S. aureus* producing Bap. These results confirmed that Gp96 serves as a ligand for native Bap in intact bacteria. Third, the specificity of the Gp96binding to Bap was further validated by the ability of polyclonal anti-Bap antibodies to block the binding of Gp96 to Bap producing bacteria (Figure 4B).Fourth, as Bap homologues have been identified in several staphylococcal species including *S. epidermidis, S.chromogenes* and *S. hyicus* [29], we investigated whether Bap homologous proteins were also able to interact with Gp96. For that, live intact *S. epidermidis* C533, *S. hyicus* 12and *S. chromogenes* C483 bacteria were incubated with recombinant Gp96 protein and binding of Gp96 was detected by western-blot using anti-Gp96. As shown in Figure 4C, all the coagulase negative staphylococcal strains producing Bap proteins were capable of pulling down Gp96 indicating that all Bap homologous proteins produced by different coagulase negative staphylococcal species interact with Gp96.

### Bap mediated adhesion to epithelial cells is independent of the presence of Gp96

**Figure 5:**
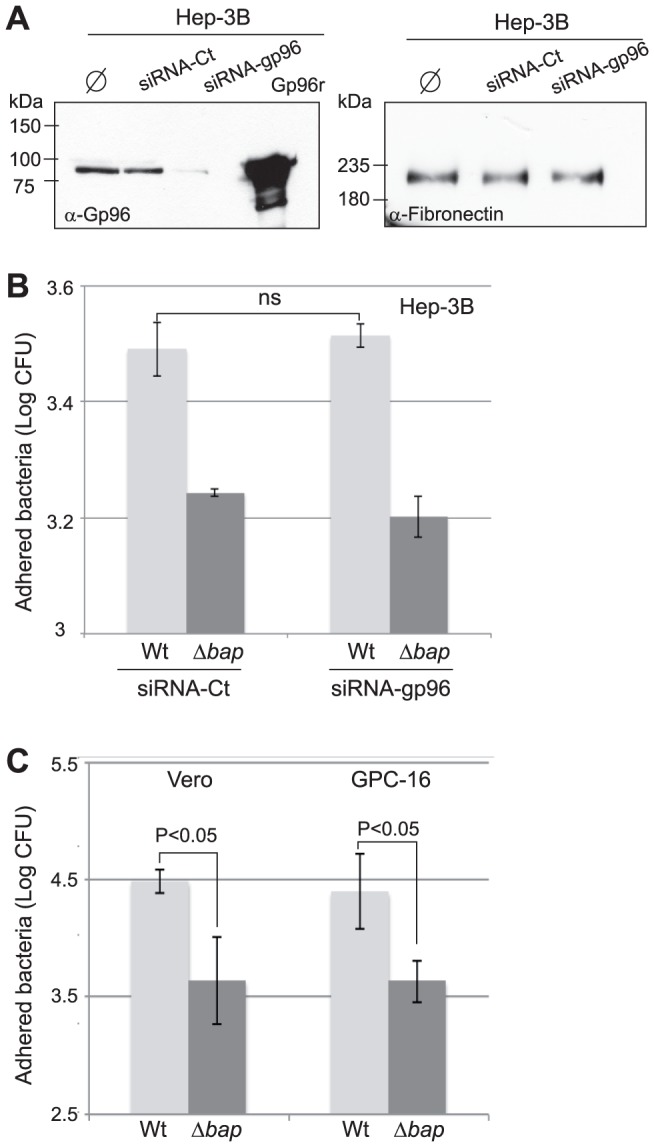
Bap mediated adhesion is independent of Gp96. A) Hep-3B cells were transfected with control (siRNA-Ct), silencer gp96 (siRNA-gp96) or nontransfected (φ) for 18 h. Gp96 expression was analyzed by western-blot using anti-Gp96 antibodies (α-Gp96) followed by signal quantification with ImageJ software. Right panel shows the levels of fibronectin expression of transfected cells. Adhesion of *S. aureus* V329 wild type (Wt) and Bap deficient strain (Δ*bap*) on Hep-3B cells transfected with control (siRNA-Ct) or silencer gp96 (siRNA-gp96) (B) or to Vero and GPC-16 cells that do not express Gp96 at the cell surface (C). After 1 h of infection, cells were lysed and cell extracts were vigorously vortexed for 2 min. Bacterial adhesion was measured by CFU counts. Error bars represent the standard deviation of at least 3 independent experiments.

To investigate whether the interaction of Bap with Gp96 was responsible for the Bap-mediated adhesion of *S. aureus* to epithelial cells, we measured the adhesion of *S. aureus* to Hep-3B cells after knocking down the Gp96 message by siRNA. The efficiency of Gp96 down regulation was determined by densitometry of Gp96 immunoblots using anti-Gp96 antibodies. As is shown in figure 5A, transfection of Gp96 specific siRNA duplexes consistently resulted in a 90% reduction in Gp96 protein levels compared to the nontransfected cells or to the cells transfected with control siRNA. The specific silencing of Gp96 expression was further confirmed by the lack of effect of Gp96 siRNA on the fibronectin protein. However, inhibition of Gp96 expression had no significant effect on the adhesion of Bap producing bacteria, suggesting that Gp96 was not required for Bap-mediated adhesion (Figure 5B).Additional evidence that Gp96 was not required for *S. aureus* adhesion to epithelial cells was obtained by assessing the adhesion of *S. aureus* V329 and Δ*bap* to the Gp96 deficient cell lines, Vero and GPC-16. The results revealed that the V329 strain still adhered significantly more efficiently (P<0.05) than the Δ*bap* mutant to both cell lines, indicating that the interaction of Bap with Gp96 was not required for the Bap-mediated adhesion to epithelial cells (Figure 5C).

### Bap and Gp96 interaction inhibits the entry of *S. aureus* into epithelial cells

**Figure 6:**
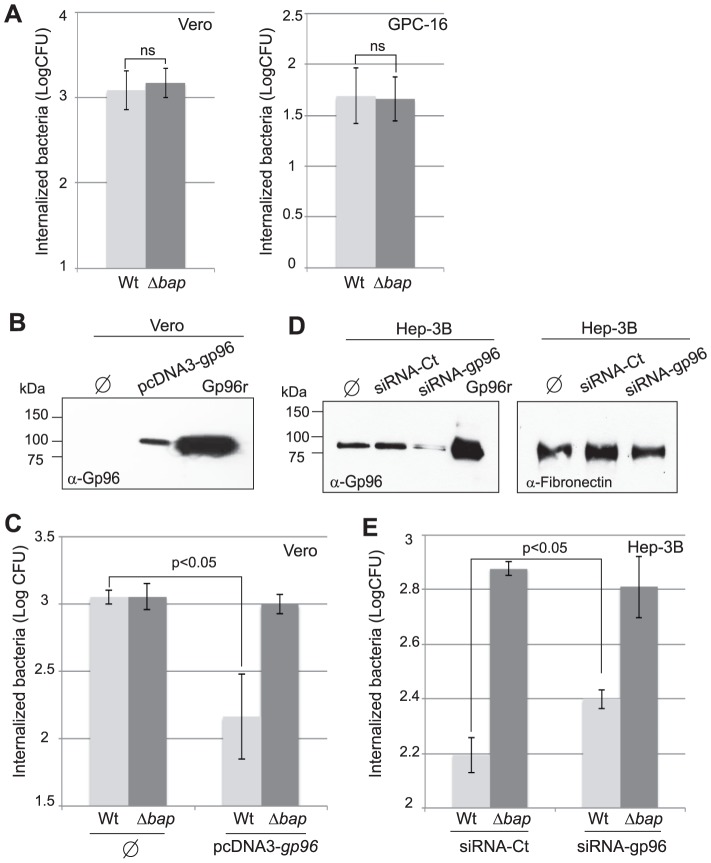
Bap-Gp96 interaction reduces the entry of *S. aureus* to epithelial cells. A) Invasion of *S. aureus* V329 wild type (Wt) and Bap deficient strain (Δ*bap*) into naturally Gp96-depleted Vero and GPC-16cells. Bacterial invasion was measured by CFU count after 2 h of gentamicin treatment. Experiments were repeated in triplicate four times for each cell line. B) Vero cells were transfected with the pcDNA3 vector containing *gp96* DNA. Expression of Gp96 was determined by western-blot analysis of cell surface proteins. Surface exposed proteins were labelled with sulpho-N-hydroxysuccinimide (NHS) biotin and purified using streptavidin columns. Captured proteins were separated in 10% SDS gel, transferred onto a nitrocellulose membrane and probed with anti-Gp96 (α-Gp96) serum. C) Entry of *S. aureus* V329 wild type and Δ*bap* mutant to Vero nontransfected cells (φ) and transfected with pcDNA3*gp96* was analyzed by gentamicin assay. Bacteria entry was measured by CFU count after 2 h of gentamicin treatment. D) Silencing Gp96 increases *S. aureus* V329 invasion. Hep-3B cells were transfected with 25 nM of control siRNA (siRNA-Ct), gp96 siRNA (siRNA-*gp96*) or nontransfected (φ). Gp96 expression was analyzed by western-blot using anti-Gp96 antibodies (α-Gp96) followed by signal quantification with imageJ software. Right panel shows the levels of fibronectin expression of transfected cells. E) Hep-3B cells transfected with control siRNA-Ct and siRNA-*gp96* were examined for invasion of *S. aureus* V329 and Bap deficient strain (Δ*bap*). Bacterial invasion was measured by CFU count after gentamicin assay. Error bars represent the standard deviation of at least 3 independent experiments.

We next examined a plausible role of the interaction of Bap with Gp96 in the invasion of *S. aureus* into epithelial cells. For that, we first determined the entry of *S. aureus* V329 and Δ*bap* to the Gp96 deficient cell lines, Vero and GPC-16. The results showed no significant differences between bacteria expressing Bap and the Δ*bap* mutant (Figure 6A), suggesting that the presence of Gp96 on the cell membrane is necessary for Bap-mediated inhibition of cell invasion. To confirm this, we carried out invasion assays on Vero cells producing Gp96 from a pcDNA3 vector containing *gp96* cDNA [56] (Figure 6B).The presence of Gp96 significantly reduced (P<0.05) the capacity of *S. aureus* V329 (Bap+) to invade the cells whereas it did not have any significant effect on the entry of Δ*bap* deficient bacteria(Figure 6C).These results confirmed that it is the interaction of Bap with Gp96 and not the fact that Bap producing bacteria are merely coated in a matrix, which reduces the capacity of *S. aureus* to invade epithelial cells. Finally, we determined the entry of *S. aureus* in Hep-3B cells after knocking down the expression of Gp96 by siRNA (Figure 6D). The number of intracellular *S. aureus* V329 increased significantly (P<0.05) when the expression of Gp96 was inhibited(Figure 6E). Taken together, these results indicate that the interaction of Bap with Gp96 inhibits the entry of *S. aureus* into epithelial cells.

### Bap-Gp96 interaction interferes with FnBPs mediated entry of *S. aureus* in epithelial cells

**Figure 7:**
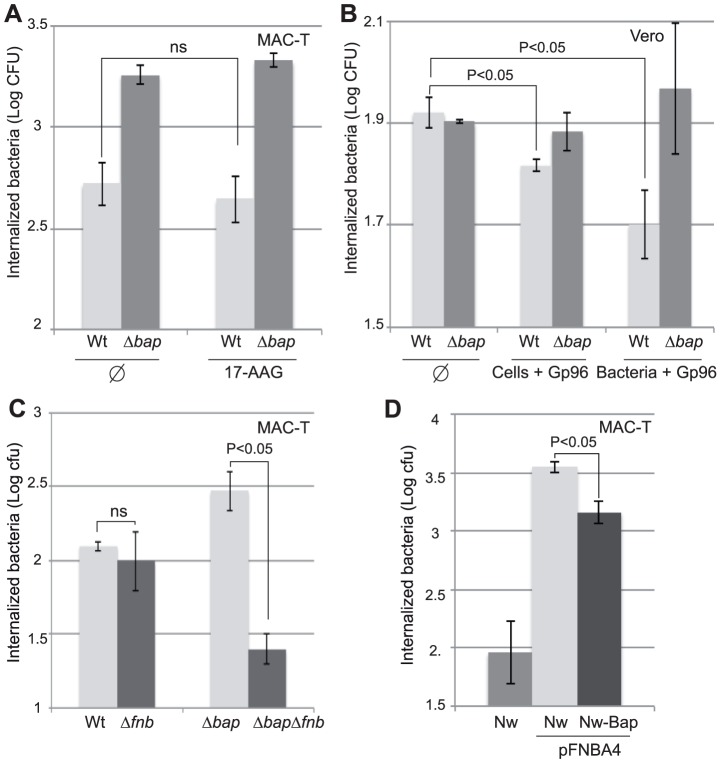
The expression of Bap minimized *S. aureus* cell entry by interfering with the FnBPs. A) Evaluation of the effect of 17-AAG on the invasion phenotype mediated by Bap. MAC-T cells were treated overnight with 1 µmol/l of 17-AAG, an inhibitor of HSP90. Invasion assays were then carried out with either *S. aureus* V329 and Δ*bap* strain. Internalized bacteria were determined by CFU count after gentamicin assay. B) Transcomplementation of Vero cells with Gp96. Invasion assay with *S. aureus* V329 and Δ*bap* was performed in Vero cells (φ), in Vero cells incubated with 10 µg/ml of recombinant Gp96 for 30 min before infection (Cells+Gp96) or with bacteria pre-incubated with 5 µg/ml of recombinant Gp96 (Bacteria+Gp96). C)Inhibition of bacterial invasion mediated by Bap requires the FnBPs. Invasion of *S. aureus* V329 wild type (Wt), a mutant in FnBPs (Δ*fnb*), Δ*bap* and double mutant Δ*fnb*Δ*bap*. After 1 h infection invasion values were calculated as the number of bacteria that survived to 2 h of gentamicin treatment in MAC-T cells.(D) MAC-T cells invasion with *S. aureus* Newman (Nw) and Newman_Bap complemented with plasmid pFNBA4 that expresses the *fnbA* gene. Experiments were performed in triplicate and repeated three times.

We next investigated the mechanisms by which the Bap-Gp96 interaction was inhibiting the entry of *S. aureus* into the host cell. To this end, we first analyzed whether Bap-Gp96 interaction was affecting the signaling pathway downstream Gp96.For that, we blocked Gp96activityand therefore the downstream signaling pathway by incubating the cells overnight in the presence of 17-AAG (17-(Allylamino)-17-demethoxydeldanamycin), which binds with high affinity into the ATP binding pocket of Gp96. The results revealed that the presence of 17-AAG (1 µM) did not affect *S. aureus*V329 entry (Figure 7A). To confirm these results and also to rule out the possibility of an indirect effect of the Gp96 absence on the expression of other Bap receptors we transcomplemented Gp96 deficient cells with purified soluble Gp96 and analyzed the infection rates of *S. aureus* V329 and Δ*bap*. Addition of soluble Gp96 prior to infection significantly reduced the entry of *S. aureus* V329 into Vero cells and this reduction was even higher when bacteria were preincubated with recombinant Gp96 before infection (Figure 7B). In contrast, the entry of the Bap deficient strain was not affected by the preincubation with Gp96 (Figure 7B). Together, these results indicate that the inhibition of *S. aureus* entry caused by Bap-Gp96 interaction is neither due to an interference with the Gp96 signaling pathway nor to a blockage of Bap binding to other cell receptors.

In cell culture models, invasion of non-professional phagocytic cells by *S. aureus* depends on the presence of FnBPs on the bacterial surface, and fibronectin and integrins on the host cell. FnBPs are important not only for adhesion but also for activating host-cell cytoskeletal remodeling via integrin-coupled signaling [57]–[63].We thus investigated the hypothesis that Bap-Gp96 interaction might be somehow interfering with the FnBPs-mediated invasion process. To this end, we deleted both *fnbA* and *fnbB* genes(Δ*fnb*) in both *S. aureus* V329 and in the Δ*bap* mutant. As shown in figure 7C, the wild type strain and its mutant in *fnbAB* showed a similar infection rate, whilst deletion of FnBPs counteracted the increased invasion capacity shown by the *S. aureus* V329 Δ*bap*. To further explore this question, we made use of *S. aureus* Newman strain that is deficient in the production of FnBPs [64] and consequently presents a very low invasion rate. As expected, complementation of this strain with plasmid pFNBA4 that expresses the *fnbA* gene significantly enhanced its entry capacity into MAC-T cells. Notably, when the *bap* gene was expressed from the chromosome of this *fnbA* complemented strain, the invasion rate decreased significantly (Figure 7D). To confirm the requirement of Gp96 in the masking effect on FnBPs-mediated internalization process activity, similar experiments were carried out in Vero cells transfected with pcDNA3*gp96*.Again,reductionof FnBPs-mediated invasion of Bap producing bacteria only occurred when Vero cells were producing Gp96 (Figure S2). Taken together, these results suggest that Bap-Gp96 interaction interferes with FnBPs-mediated entry of *S. aureus* in epithelial cells.

### A shorter allele of Bap exhibits a low capacity to inhibit FnBPs-mediated bacterial invasion

**Figure 8:**
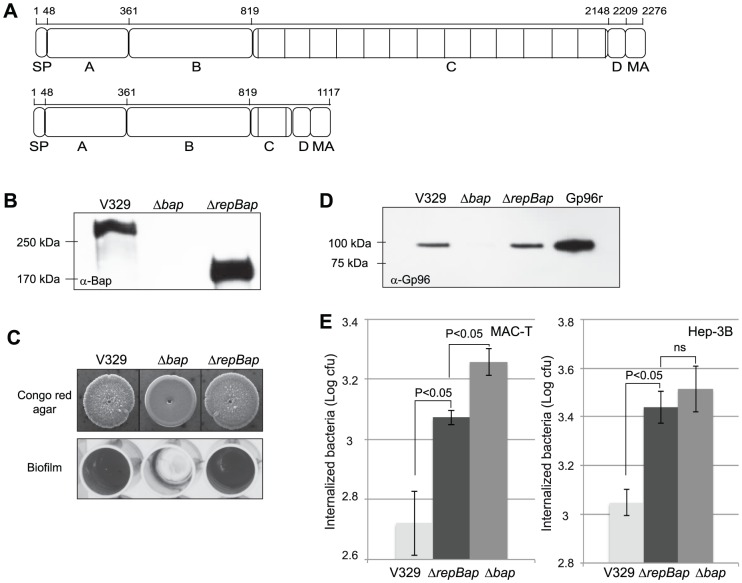
Effect of Bap repetitions in Gp96 interaction and cell invasion. A) Structural organization of Bap with or without repetitions. SP, signal peptide; A, region A; B, region B; C, repetition region; D, region of serine-aspartate (SD) repeats; MA cell wall anchor. B) Western-blot analysis with anti-Bap (α-Bap) serum of surface protein preparations obtained under isosmotic conditions from *S. aureus* V329 (Bap+), Δ*bap* and Δ*repBap* (truncated Bap). C) Biofilm phenotypes of *S. aureus* V329, Δ*bap* and Δ*repBap*. Congo red morphology on congo red agar plates and biofilm formation in microtiter plates. D) Interaction of Bap-truncated protein with Gp96. *S. aureus* V329 (Bap+), Δ*bap* and Δ*repBap* strains were subcultured with 5 µg/ml of recombinant Gp96. Unbound Gp96 was removed by extensive washing and Gp96 bound to bacteria was detected using immunoblot analysis. E) Entry of *S. aureus* V329, Δ*bap* and Δ*repBap* strains was analyzed by gentamicin assay in MAC-T and Hep-3B cells. Experiments were performed in triplicate and repeated three times for each cell line.

As Bap is a large protein of 2276-amino-acid we wondered whether the interaction of Bap with Gp96 could act as a steric hindrance, limiting the accessibility of FnBPs to its target, fibronectin, and in consequence minimizing cell entry. To further assess this hypothesis we constructed a *S. aureus* strain that produces a recombinant short Bap protein, containing a single repetition (ΔrepBap), and leading to a Bap derivative about half the size of the wild type protein (Figure 8A). Western-blot analysis using anti-Bap antibodies confirmed that *S. aureus* ΔrepBap strain produced similar levels of the short Bap protein compared to wildtype strain (Figure 8B). Moreover, the biofilm formed by *S. aureus* ΔrepBap strain was indistinguishable from that produced by V329 strain indicating that the short Bap variant is functional (Figure 8 C).

Then, we investigated whether ΔrepBap still retained the capacity to interact with Gp96 using the pull-down assay. As shown in figure 8D, Gp96 was pulled down by intact cells producing ΔrepBap as efficiently as bacteria producing wildtype Bap, indicating that a Bap allele containing a single repetition can interact with Gp96. *S. aureus* ΔrepBap strain was then tested for its ability to invade epithelial cells. The efficiency of entry of *S. aureus* producing a ΔrepBap-mediated biofilm matrix was significantly higher than that of wildtype bacteria and very similar to that of the Bap deficient strain (P<0.05) (Figure 8E). Together these data indicate that a short version of Bap, although still able to interact with Gp96, is unable to block the infection capacity of wild type bacteria, and thus support the hypothesis that the interaction of Bap with Gp96 might cause a stearic impediment that interferes with the FnBPs binding to fibronectin.

### Bap hinders *S. aureus* mammary gland invasion in a lactating mouse mastitis model

**Figure 9:**
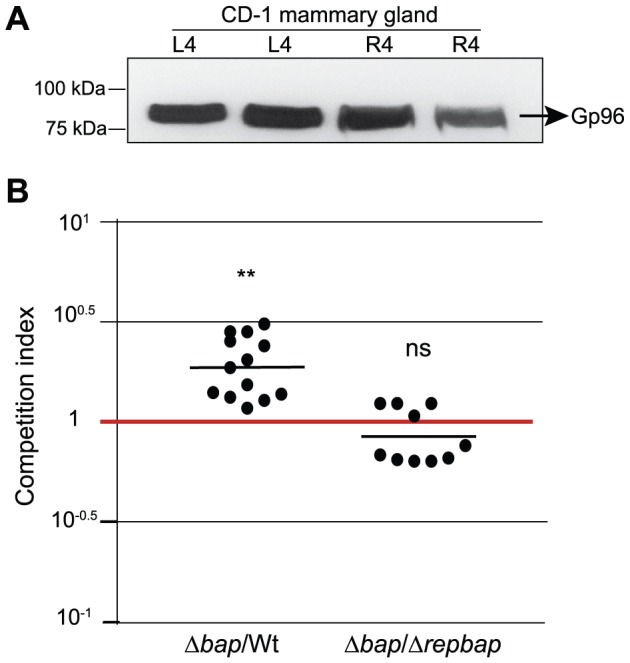
Effect of Bap expression in *S. aureus* invasion into mammary glands using a murine mastitis model. A) Gp96 expression in mammary glands L4 and R4. Glands were homogenized and proteins were resolved by 10% SDS-polyacrylamide gels. Gp96 was detected using anti-Gp96 antibodies. B) Competition indexes of Δ*bap*-Wt and Δ*bap*-Δ*repBap*. Mice mammary glands were coinfected with equal numbers of the strains tested. After 18 h post-infection mammary glands were treated (intra-mammary administration) with PBS with gentamicin for 3 h. Bacterial counts (CFU) were obtained from each mammary gland. Competition indexes were calculated and statistical differences were determined with a t-test. Horizontal bars represent the means of the competition indexes. Asterisks indicate that competition indexes are significantly greater than one (**, P<0.01 [very significant]). ns, non significant differences.

To investigate the relevance of the Bap-dependent biofilm matrix in the prevention of *in vivo* host cell invasion we used a lactating mouse mastitis model. We first confirmed that Gp96 is expressed in mammary glands of lactating mice by western-blot using anti-Gp96 antibodies (Figure 9A). Two mammary glands (L4 and R4) of a group of 7 lactating mice were inoculated with a bacterial solution containing 10^6^ CFU of *S. aureus* V329 (Bap+) and *bap::tet* strains. After 18 h post-infection mammary glands were treated 3 h with a solution of PBS containing gentamicin to remove all the extracellular bacteria. Then, the mammary glands were removed aseptically, homogenized and several dilutions were plated on selective agar. To evaluate the invasion capacity of each strain we used the competition index method. As a previous control, we first verified that the invasion differences detected *in-vitro* between the wild-type and the *bap*-deficient strain were maintained when both strains were used to co-infected MAC-T cells (Figure S3).In agreement with *in vitro* assays, wild type bacteria producing Bap showed a significantly lower capacity to invade the mammary gland cells compared with Bap deficient bacteria (Figure 9B). Next, the experiment was repeated comparing the *in vivo* invasion capacity of Bap deficient bacteria and the strain expressing a short Bap derivative. These two strains showed a very similar capacity to invade mammary gland cells (Figure 9B). These results again confirmed *in vitro* results showing that the entry efficiency of *S. aureus* producing a ΔrepBap-mediated biofilm matrix is equal to that of the Bap deficient strain. Together, these results strongly suggest that full-size Bap acts as anti-invasion factor of the mammary gland *in vivo*.

## Discussion

Biofilm formation is recognized as causing or exacerbating several *S. aureus* chronic infections such as osteomyelitis, endocarditis and device related infections. The molecular mechanisms underlying the persistence of biofilm infections have been mainly associated with the protection barrier that the biofilm matrix provides against the host immune response and the antibiotic treatments to the embedded bacteria. For example, exopolysaccharidic PIA/PNAG biofilm matrix protects *S. aureus* from phagocytosis by polymorphic neutrophils and from antibodies mediated opsonisation and retards the rate of antibiotic penetration enough to induce the expression of genes that mediate resistance [32], [65]–[67].

With the aim to identify new functions for the biofilm matrix that may facilitate the development of persistent infections, we have investigated the role of the proteinaceous Bap-mediated biofilm matrix in the interaction with host cells. Our results provide evidences that the Bap biofilm matrix promotes the adhesion of *S. aureus* to different types of epithelial cells. For this function, Bap does not require the participation of any other staphylococcal factors, because production of Bap in *E. faecalis* is sufficient to bestow the capacity to adhere to epithelial cells. Screening for the specific molecular target on the epithelial cells using a ligand immunoblot overlay approach revealed that Bap binds to Gp96, also known as GRP94 or endoplasmin, which is a major chaperon of the lumen of the endoplasmic reticulum (ER) [42], [43], [68]. We initially received this result with caution because, using the same approach, Gp96 had been previously identified as the ligand for Vip, a surface protein of *Listeria monocytogenes* without homology with Bap [52]. To exclude that Bap might be interacting with Gp96 in an artificial way, confirmatory pull down studies were carried out. The results revealed that (i) recombinant Gp96 was pulled down with native Bap protein anchored to the cell wall of intact bacteria; (ii) recombinant Gp96 was pulled down with soluble Bap and anti-Bap antibodies; and (iii)anti-Bap antibodies were able to inhibit the interaction between Bap and Gp96. Another caution about Gp96 being the ligand for Bap was that Gp96 was initially described as an endoplasmic reticulum (ER) protein based on the presence of the KDEL motif in the carboxy-terminal domain of the protein [47], [69], [70]. However, in agreement with other studies [45]–[50], [52], [54], [55], we have found that Gp96 is not restricted to the ER and is also present on the surface of some epithelial cell lines (Hep-3B and MAC-T).

Given that Bap was interacting with Gp96 and promoting adherence to epithelial cells, we hypothesized that binding of Bap to Gp96 might be responsible for the enhanced adherence of Bap producing bacteria to the surface of the epithelial cells. In contrast to our assumption, we found that depletion of Gp96 expression by siRNA in Hep-3B cells did not reduce the adhesion capacity of Bap positive bacteria. Furthermore, bacteria producing Bap also displayed a higher capacity to adhere to cells that do not produce Gp96 than Bap deficient bacteria, indicating that Bap was interacting with another factor, different from Gp96, to promote the adhesion to the epithelial cells. These results raised the question as to why this receptor was not identified with the ligand overlay experiment. At least two reasons can be envisioned to explain this failure. Either the ligand is not a protein and/or Bap might recognize its ligand only in its native folded structure. Further experiments will be needed to identify this additional cellular ligand.

On the other hand, we have shown that the interaction of Bap with Gp96 inhibits bacterial entry into epithelial cells. Several results support this conclusion: (i) Bap deficient bacteria showed higher levels of invasion than the corresponding wildtype strain in cells producing Gp96; (ii) the invasion differences between Bap positive and negative strains disappeared when invasion was tested inGp96 negative cells; (iii) the entry of Bap positive bacteria decreased in cells overproducing Gp96 and increased in cells depleted of Gp96 by the expression of siRNA; (iv) preincubation of cells or bacteria with soluble Gp96 inhibited the *S. aureus* entry into the cells. These former results also indicate that Bap does not need to interact with membrane anchored Gp96 to inhibit bacterial entry. How does the interaction between Bap and Gp96 interfere with *S. aureus* invasion? We initially explored the possibility that the binding of Bap with Gp96 might interfere with the signaling pathway downstream Gp96. Against this hypothesis, we have shown that treatment of MAC-T and Hep-3B cell with 17-AAG, a drug that affects the signaling pathway regulated by Gp96, does not affect the invasion levels of Bap producing bacteria. Furthermore, incubation of Vero cells with soluble Gp96, which is not linked with the cytoplasmic partners, still reduced the capacity of *S. aureus* to invade the cells. Alternatively, the interaction of Bap with Gp96 could be directly interfering with the recognition of another host ligand. The main known mechanism of *S. aureus* invasion into the host cell is mediated by the bacterial fibronectin binding proteins, FnBPA and FnBPB and host cell fibronectin andα_5_β_1_ integrins [57]–[63].Our results suggest that the interaction of Bap with Gp96 interferes with the FnBPs-fibronectin-integrin invasion pathway because deletion of FnBP proteins counteracted the increased invasion rates of Bap deficient bacteria. In addition, overexpression of FnBPs restored the invasion capacity of Bap negative bacteria but did not change the decreased invasion capacity of Bap producing bacteria. It is worth noting that the interference of the FnBPs invasion pathway depends on the length of Bap, because a short but still functional allele of Bap displayed a significantly lower efficiency than the full-length protein to inhibit bacterial entry.

Gp96 has been reported to be the ligand for various bacterial surface proteins, though the consequences of this interaction seem to be different depending on the bacteria [52], [53], [55], [56], [71]–[73]. The bacterial Outer membrane protein A (OmpA) of *Escherichia coli* interacts with Ecgp96, an homolog of Gp96 that is highly expressed in brain microvascular endothelial cells during meningitis infection, and induces bacterial invasion [56], [71], [72]. Also, Cabanes et al. [52]identified that the interaction of Vip with Gp96 promotes *Listeria monocytogenes* invasion of the cell. Gp96 has also been described to interact with other bacterial products such as the exotoxin A (TxA) of *Clostridium difficile* [74]and the outer membrane vesicles (OMVs) produced by adherent-invasive *E. coli* (AIEC) promoting bacterial invasion [55]. In contrast, the interaction of the outer membrane porin PorB_IA_ of *Neisseria gonorrhoeae* with Gp96 inhibits bacterial invasion [53]. Moreover, the interaction of Hsp90 (a chaperone homolog to Gp96) with the trimeric surface protein NadA also interferes with bacterial adhesion and invasion [73]. It is worth noting that OmpA, Vip, PorB and NadA do not show any homology with Bap that would explain the interaction with the same ligand. One may speculate that Gp96 is able to bind unspecifically to these bacterial proteins due to its chaperone structure. However, pull down and ligand overlay assays with bacteria producing two other members of the Bap family (Esp protein of *Enterococcus faecalis* and BapA from *Salmonella* Enteritidis) or an unrelated bacterial protein (dispersin)did not show any interaction with Gp96 (data not shown), excluding the hypothesis that Gp96 can interact with any bacterial protein. Also, these results indicate that not all the members of the Bap family can interact with Gp96 protein.

The presence of Bapin *S. aureus* is specifically enriched, by yet unknown reasons, in mastitis-derived isolates where Bap facilitates the persistence of the bacteria in lactating ewes mammary glands [30]. Bap homologous proteins are also encoded in different coagulase negative staphylococcal species associated with chronic mastitis infections [75]–[77].Gp96 is expressed in the bovine mammary glands during the lactating period (Figure 9A) and its presence in the milk has been suggested to be a host defense mechanism [78]. In the present study, we have found a connection between these two findings using a lactating mice infection model, where the Bap expression had a profound impact on the capacity of the bacteria to adhere and invade the mammary gland epithelial cells. Overall, our results support the view that Bap mediated biofilm development facilitates the formation of bacterial aggregates that survive attached to the epithelial cells of the mammary gland by impairing the bacterial internalization through the interaction with Gp96. In this situation, the Bap biofilm matrix promotes the establishment of long-term persistent infections and mediates immune evasion by masking surface antigens. Whether the proteinaceous Bap-dependent biofilm matrix of different bacterial species is playing a similar role in the interaction with the host cells is worthy of further exploration.

## Materials and Methods

### Ethics statement

All animal studies were reviewed and approved by the “Comité de Etica, Experimentación Animal y Bioseguridad” of the Universidad Pública de Navarra (approved protocol PI-6/10). Work was carried out at the Centro de Agrobiotecnología building (Idab) under the principles and guidelines described in the *“European Directive 86/609/EEC”* for the protection of animals used for experimental purposes.

### Bacterial strains, cell culture and culture conditions

*Escherichia coli* DH10B was cultured in Luria-Bertani (LB) media or on LB agar (Pronadisa, Spain) with appropriate antibiotics. Staphylococcal strains were grown in Trypticase soy broth (TSB) or Trypticase soy agar (TSA) supplemented with glucose 0.25% (TSB-glu) when indicated. Media were supplemented with the following appropriate antibiotics at indicated concentrations: erythromycin 20 µg ml^−1^ or 1.5 µg ml^−1^, chloramphenicol 20 µg ml^−1^, ampicillin 100 µg ml^−1^, tetracycline 10 µg ml^−1^.Bovine mammary gland epithelial cell line (MAC-T) was used because *bap* gene is frequently present in *S. aureus* isolates causing mastitis infections. Human hepathoma cell line (Hep-3B, ATCC number CCL-2) was used as an example of any other cell line different from mammary gland epithelial cells. Vero epithelial cells (ATCC number CCL-81) and guinea–pig GPC-16 epithelial cells were used due to their low extracellular Gp96 expression [52]. Cells were maintained in Dulbecco's modified Eagle's medium (DMEM) (Gibco-BRL) supplemented with 10% heat-inactivated fetal bovine serum (Gibco-BRL).

### Manipulation of DNA

To generate the deletion in the *bap* gene we amplified by PCR two fragments of 500 bp that flanked the left sequence of the gene using primers Δbap-A (ggatccgacatacattagatatttgg) and Δbap-B (ctcgagcaattttatgacgcactatt) and the right sequence of *bap* using primers Δbap-C (ctcgagcccattttattattggttctg) and Δbap-D (gaattcgccgaaatgttggccgtattc). Fragments were then fused by ligation into the shuttle vector pMAD, and the resulting plasmid was transformed in V329 strain by electroporation. Allelic exchange in the absence of a selection marker was performed as previously described [27]. FnBPs mutant in V329 and Δ*bap* strains was performed as previously described [17]. To generate the Δ*repBap* strain, the *bap* gene of *S. aureus* V858 whose *bap* contains a single C-repeat was amplified using DNA polymerase KOD XL (Merck) with primers BapXho-5 (ctcgagtaaaaaaatttattttgaggtgag) and BapXho-3 (ctcgagctctccacctttgtaagtg). The gene was cloned into the pMAD plasmid and the resulting plasmid was transformed into Δ*bap* strain by electroporation. Allelic exchange in the absence of a selection marker was performed as previously described [27]. Δ*repBap* strains were verified using primers Bap-6m (cctatatcgaaggtgtagaattgcac) and Bap-7c (gctgttgaagttaatactgtacctgc).

*S. aurues bap*::tet strain was constructed using plasmid pJP188 [79].For complementation experiments, the multicopy plasmid pFNBA4 [80] that carries the wild-type *fnbA* gene of *S. aureus*8325-4 was used. *E. faecalis* 23 was complemented with plasmid pBT2-bap.

### Generation of purified Bap recombinant protein

For Bap purification we constructed a recombinant Bap protein in which the LPXTG motif of Bap was replaced by the 6-histidine tag. For that, a 281 bp fragment upstream the LPXTG was amplified using primers BapEco-A (gaattcaattcaggtgctggagacac) and BapBam-B (ggatcctcagtggtggtggtggtggtgttctggtaattcattttg). A 600 bp fragment downstream Bap was amplified using primers BapBam-C (ggatccatgtttaaattattgtaaat) and BapEco-D (gaattcgccgaaatgttggccgtattc). Fragments were cloned into the EcoRI site of the pMAD plasmid [81]and the resulting plasmid was transformed into V329 strain by electroporation using a previously described protocol [30]. The construction was verified by sequencing the insert. Allelic exchange in the absence of a selection marker was performed as previously described [27]. As the LPXTG motif was replaced by the 6xHis tag, the recombinant Bap-6xHis protein was obtained at the supernatant. One liter of the supernatant was concentrated using centrifugal filter units 10,000 wco (Millipore). Bap-6xHis tagged protein was purified from the supernatant using His GraviTrap affinity columns (GE Healthcare).

### Ligand overlayer assay

MAC-T and Hep-3B epithelial cells were lysed in RIPA-buffer (150 mM NaCl, 50 mM Tris pH7.5, 0.1% SDS, 1% Triton X-100, 0.5% sodium deoxycholate, 1 mM sodiumorthovanadate, 10 mM NaF, β-glycerophosphate 100 mM and protease inhibitor cocktail (Roche)). Lysates were clarified and protein concentration was determined. 40 µg of cell lysates were resolved by 10% sodium dodecyl sulphate (SDS)-polyacrylamide gel electrophoresis. Proteins were transferred onto a nitrocellulose membrane and blocked overnight. The membrane was then incubated with 50 µg/ml of purified Bap, washed and incubated with anti-bap antibodies diluted 1∶20,000 [30]. Alkaline phosphatase-conjugated goat anti-rabbit immunoglobulin G diluted 1∶10,000 was used as secondary antibody. Protein identification was analyzed by MALDI-TOF analysis followed by peptide mass fingerprinting [16].

### Pull-down assays

10 µg of the recombinant Gp96 protein (SPP-766 Stressgen) were mixed with 10 µg of purified Bap. The mix was incubated at 4°C for 2 h in slow agitation. Gp96 was immunoprecipitated using 1.5 µl of anti-Bap antibodies, for 2 h at 4°C and then with 50 µl of Protein G sepharose beads (GE Healthcare). Immunoprecipitated proteins were boiled in Laemmli buffer and analyzed by SDS-polyacrylamide gel electrophoresis, inmmunoblotted with primary antibodies anti-Bap (1∶2500) or anti-Gp96 (1∶1000)monoclonal antibodies (SPA-850 Stressgen)and with secondary antibodies goat anti-rabbit immunoglobulin-G HRP (Thermo) or anti-rat immunoglobulin-G HRP (SAB-200 Stressgen). Pull down experiment using bacteria was performed as follows. *S. aureus* V329 expressing Bap, Δ*bap*, Δ*repBap* and CNS(*S. epidermidis* C533, *S. hyicus* 12, and *S. chromogenes* C483) were grown overnight at 37°C. A volume of cells corresponding to an OD_600_ value of 5 was centrifuged and washed twice with PBS buffer. Bacteria were then incubated with 5 µg/ml of recombinant Gp96 protein in the absence or in the presence of anti-Bap antibodies for 2 h at 4°C and slow agitation. Unbound Gp96 was removed by washing the bacteria 4 times with 1 ml of PBS buffer. Immunoprecipitated Gp96 was detected using anti-Gp96 antibodies and anti-rat immunoglobulin-G HRP.

### Biofilm phenotype

Biofilm formation assay in microtiter wells was performed as described [18]. Briefly, strains were grown overnight at 37°C and were diluted 1∶40 in TSB-gluc. Cell suspension was used to inoculate sterile 96-well polystyrene microtiter plates (IWAKI). After 24 hours at 37°C wells were gently rinsed three times with water, dried and stained with 0.1% of crystal violet for 15 min. Colony morphology of *S. aureus* was analyzed using Congo red agar plates [82]–[84].Congo red agar was prepared as follow: 30 g/l of trypticase soy (Pronadisa), 15 g/l of agar (Pronadisa), 0.8 g/l of Congo red stain (Sigma) and 20 g/l of sucrose. The Congo red stain and the sucrose solution were autoclaved separately (121°C for 20 minutes) and (115°C for 15 minutes) respectively. *S. aureus* strains were streaked on congo red agar and were incubated at 37°C for 24 hours. Rough colonies are being indicative of biofilm formation.

### Epithelial cell adhesion and invasion assays

Adherence and invasion experiments were performed as described previously [57]. Briefly, prior to use, wells were seeded with 0.3×10^6^ cells in 6-well tissue culture plates and 0.5×10^5^ cells in 24-well tissue culture plates. Once cells were confluent (1.2×10^6^ or 0.2 10^6^ cells per well) the culture medium was removed and cells were washed with DMEM plus 10% heat-inactivated fetal bovine serum. For adherence assays, overnight bacterial cultures were mixed vigorously and added to the monolayer cells in a multiplicity of infection of 10 in DMEM. Incubation was carried out 1 hour at 37°C in 5% CO_2_. To remove non-adherent bacteria, cells were washed three times with sterile PBS. Eukaryotic cells were lysed with 0.1% Triton X-100. Before plating extracts were mixed vigorously by vortexing and sonication. The number of adherent bacteria were determined by serial dilution and plating. For invasion assays, bacteria were added to the monolayer cells in a multiplicity of infection of 40 in DMEM. Incubation was carried out for 1 hour at 37°C in 5% CO_2_. To kill extracellular bacteria, media was replaced with 2 ml of DMEM containing 50 µg ml^−1^ of gentamicin (SIGMA) for 2 hour. Cell monolayers were washed three times with sterile PBS and lysed with 0.1% Triton X-100. Before plating extracts were mixed vigorously by vortexing and sonication. The number of intracellular bacteria was determined by serial dilution and plating. Experiments were performed in triplicate.

### Cell surface protein biotinylation

Cell surface biotinylation was performed using Pierce Cell surface protein isolation kit according to the manufacturer's protocol. Briefly, 4 flasks of 75 cm^2^ of live confluent cells were incubated with Sulfo-NHS-SS-Biotin for 30 min at 4°C. Sulfo-NHS-SS-Biotin was quenched and biotinylized cells were lyzed. For isolation of labelled proteins lyzed cell were incubated with NeutrAvidin Agarose. Eluted proteins were resolved by 10% sodium dodecyl sulphate (SDS)-polyacrylamide gel electrophoresis. Immunodetection was performed following protein transfer onto nitrocellulose membrane and incubation with anti-Gp96 antibodies. To control membrane impermeability α-catenin was detected using anti-α-catenin antibodies (H-297 Santa Cruz Biotechnologies).

### Immunofluorescence analysis

Cells grown on coverslips, fixed with paraformaldehyde 3.5% (SIGMA) and stained with anti-Gp96 (H-212; Santa Cruz Biotech) diluted 1∶100 and stained secondary antibody Alexa Fluor 488-conjugated goat anti-rabbit (Invitrogen). Cells were then permeabilized (0.1% triton X-100 for 5 min in PBS). Alexa-Fluor 647-Phalloidin (Invitrogen) diluted 1∶200 was used to label actin filaments. Preparations were observed with an epifluorescence microscope and images were acquired and analyzed with EZ-C1 software (Nikon).

### Plasmid DNA and silencer *gp96* transfections

Transient transfection of pcDNA3-*gp96* in Vero cells was performed using Lipofectamine 2000 (Invitrogen) following the manufacturer's protocol. Briefly, lipofectamine was diluted 1∶25 in DMEM media. Diluted lipofectamine was then mixed with 0.8 µg of DNA in a 2∶1 ratio and the mixture was incubated for 20 min at room temperature. After incubation, 100 µl were added to a 24-well culture vessel containing Vero confluent cells. To silence gene expression by siRNA, Hep-3B cells were transfected with Gp96 silencer siRNA (Hs_TRA1_9) and control siRNA (Allstars Negative Control) purchased from Qiagen (Valencia, CA). Transfection was performed using a Ready-to-Use mix from Qiagen as described by the manufacturer. Cells were collected for immunodetection of Gp96 using monoclonal anti-Gp96 antibodies and fibronectin using anti-fibronectin antibodies (SIGMA).Gp96 expression was determined by density measurement of digital images using ImageJ software.

### Treatment with 17-AAG and transcomplementation with Gp96

MAC-T and Hep-3B monolayers were treated with 1 µM of 17-(Allylamino)-17-demethoxydeldanamycin (17-AAG) or the vehicle control DMSO, one day before the experiments. Overnight treatments of MAC-T and Hep-3B had no effect on cell morphology. Then, cells were washed and incubated with bacteria for invasion assays. Transcomplemetation of Vero cells were performed as described by Rechner et al., 2007 [53]. Briefly, 0.5×10^6^ Vero cells were incubated with 10 µg/ml of Gp96 for 30 min (cells+Gp96) and infected with *S. aureus* V329 and Δ*bap* strains (MOI = 40) to perform invasion assays. Additionally, *S. aureus* V329 and Δ*bap* strains were incubated with 5 µg/ml of recombinant Gp96 protein for 2 h at 4°C. After washing bacteria were used for infection of Vero cells (0.5×10^6^) (bacteria+Gp96).

### Mice intramammary infection model

CD1 mice were maintained in the animal facility of the Institute of Agrobiotechnology, Public University of Navarra. Seven to 10 days after parturition, pups of a group of 7 lactating female mice were removed 2 h before bacterial inoculation. A mixture of ketamine/xylazine was used to anaesthetize lactating mice. 100 µl of a solution containing 10^6^ CFU of a mix of *S. aureus bap*::tet and V329 strains or a mix of *bap*::tet and Δ*repBap* strains were used to inoculate L4 (on the left) and R4 (on the right) mammary glands. After 18 h post-infection L4 and R4 mammary glands were inoculated with 200 µl of a solution of PBS containing 100 µg ml^−1^ of gentamicin (SIGMA). After 3 h,L4 and R4 mammary glands were aseptically removed and homogenized. Viable counts were performed on these homogenates by plating the samples on TSA and TSA containing tetracycline. To evaluate the invasion capacity of each strain we used the competition index method. A competition index equal to 1 indicates similar invasiveness for both strains. A competition index significantly greater than 1 indicates a higher invasion capacity of the Bap mutant and a competition index lower than 1 indicates the opposite. Prior to the start of the coinfection assays, a competition experiment was performed with all strains tested to confirm that coincubation of the strains did not affect their growth capacity.

### Statistical analysis

Data corresponding to adhesion and invasion were compared using the Mann-Whitney tests. Competition indexes of wild type-Δ*bap* and Δ*bap-*Δ*repBap* were calculated using t-test and statistical differences were determined with the t-test.

## Supporting Information

Figure S1 Effect of the presence of PIA/PNAG exopolysaccharidic biofilm matrix in *S. aureus* invasion capacity. A) Dot-blot analysis showing PIA/PNAG accumulation in *S. aureus* V329 and Δ*bap* complemented with pSC18 and *S. aureus* ISP479r. Cell surface extracts were treated with proteinase K and spotted onto nitrocellulose filters diluted 1/10. PNAG production was detected with anti-*S. aureus* PNAG antiserum. Invasion of *S. aureus* V329, V329 pSC18, Δ*bap* mutant, Δ*bap* pSC18, ISP479r and ISP479r Δ*ica* into MAC-T (B) and Hep-3B (C) cells. Bacterial invasion was measured by CFU count after 2 h of gentamicin treatment. Statistical analyses were performed using Mann-Whitney tests.
(EPS)

Figure S2 Invasion of *S. aureus* Newman (Nw) and Newman_Bap complemented with plasmid pFNBA4 that expresses the *fnbA* gene to Vero cells and Vero cells transfected with pcDNA3*gp96*. After 1 h infection invasion values were calculated as the number of bacteria that survived to 2 h of gentamicin treatment.
(EPS)

Figure S3 Competition index of Δ*bap*-Wt and Δ*bap*-Δ*repBap* strains *in vitro*. An equal number of bacteria of *S. aureus* V329 (Wt) and Δ*bap* or Δ*bap* and Δ*repBap* were used to infected MAC-T epithelial cells. After 1 h infection, invasion efficiency was calculated as the number of cfu that survived to 2 h of gentamicin treatment. Statistical differences were determined with Mann-Whitney tests. Horizontal bars represent the means of triplicates of two independent experiments. Asterisks indicate differences in competition indexes greater than one (P<0,05); ns, non-significant differences.
(EPS)
